# Platelet factor 4 and stromal cell-derived factor are novel prognostic biomarkers for cerebral vasospasm and mortality after subarachnoid hemorrhage

**DOI:** 10.1038/s41598-025-18117-4

**Published:** 2025-09-05

**Authors:** Dilaware Khan, Igor Fischer, Sihmehmet Sahan, Michael Hewera, Katharina Faust, Sajjad Muhammad

**Affiliations:** Department of Neurosurgery, Medical Faculty and University Hospital Düsseldorf, Heinrich-Heine-University, Moorenstraße 5, 40225 Duesseldorf, Germany

**Keywords:** SAH, Predictive model, SDF, PF4, CVS, Mortality, Stroke, Cerebrovascular disorders

## Abstract

Aneurysmal subarachnoid hemorrhage (aSAH) is a life-threatening condition associated with high rates of morbidity and mortality, mainly due to post-hemorrhagic complications such as cerebral vasospasm (CVS) and delayed cerebral ischemia (DCI). Recent evidence implicates platelet activation and inflammatory mediators in the cascade of secondary injury following aSAH. Monitoring and timely treatment of post-SAH complications is critical to improve clinical outcomes. Current methods of radiological diagnostic for monitoring are laborious and bound to multiple risks, including radiation exposure. Point-of-care blood biomarkers early after SAH are urgently needed to timely detect and treat post-SAH complications. This prospectively designed cohort study aimed to search for novel predictive biomarkers for vasospasm and clinical outcome with overarching goal to improve treatment decision during intensive care treatment. In this prospective study conducted from 2020 to 2022, 63 aSAH patients (16 male and 47 female) were enrolled. Blood samples were collected to measure platelet factor 4 (PF4) and stromal cell derived factor (SDF) levels. To detect vasospasm, computed tomography (CT) perfusions at day 0, 4, 7 and 11 after the SAH was performed and mean transient time (MTT) was quantified as a measure of cerebral perfusion. Clinical outcome was analysed using modified rankin scale (mRS). Clinical and radiological data was recorded prospectively. The association between PF4 levels and CVS was assessed using receiver operating characteristic (ROC) curve analysis and logistic regression, while linear regression analyses were performed to examine the correlations of PF4 with mean transit times (MTT) and of SDF with the day of death among non-survivors. Logistic regression revealed that each 1 ng/mL increase in PF4 was associated with a 38% increase in the odds of developing CVS (OR = 1.38; 95% CI: 1.07–1.79; *p* = 0.01). Moreover, elevated PF4 levels were significantly correlated with longer MTT (β = 0.107 per ng/mL; 95% CI: 0.02–0.19; *p* = 0.02), suggesting impaired cerebral perfusion. Additionally, among non-surviving patients, higher SDF levels were significantly associated with a later occurrence of death (β = 0.01 per pg/mL; 95% CI: 0.001–0.019; *p* = 0.03). The findings indicate that PF4 and SDF are promising biomarkers in aSAH. Elevated PF4 levels are associated with an increased risk of CVS and impaired cerebral perfusion, while higher SDF levels correlate with mortality. These biomarkers may offer valuable insights for early risk stratification and pave the way for targeted therapeutic interventions in patients with aSAH.

## Introduction

Subarachnoid hemorrhage (SAH) remains one of the most devastating forms of stroke, marked by sudden bleeding into the subarachnoid space and associated with high rates of morbidity and mortality^1^. The rupture of intracranial aneurysms accounts for around 85% of the SAH events and is bound to a mortality rate of up to around 40% of cases^2^. Despite advances in neurocritical care, the morbidity and mortality are still high. The post-SAH complications, such as cerebral vasospasm and delayed cerebral ischemia, continue to contribute significantly to poor outcomes^3^. The pathophysiology of SAH involves the initial hemorrhagic insult followed by a complex cascade of secondary brain injury mechanisms, including inflammatory responses, oxidative stress, and microthrombotic events contributing to post-aSAH complications, prognosis, and outcome^4,5^. In this context, the identification of reliable biomarkers is critical for early risk stratification, timely intervention, and the development of targeted therapeutic strategies.

Activated platelets contribute to oxidative stress^6^ and inflammation^6–8^ which are known to induce vascular cell dysfunction^6,9,10^ leading to aneurysm formation and rupture^11,12^ resulting in SAH. Recent studies have highlighted the pivotal role of inflammatory and hemostatic processes in mediating secondary brain injury, complications, and clinical outcome following SAH^4,13–15^. Platelet activation is crucial in causing neuronal damage, contributing to neurodegenerative diseases^16^ and vascular dysfunction resulting in the formation and rupture of atherosclerotic lesions and aneurysms^7,8^. Platelet factor 4 (PF4) is a chemokine released from activated platelets that modulates coagulation and inflammatory pathways. Elevated PF4 levels have been implicated in vascular inflammation and the formation of microthrombi^17^ causing impaired cerebral perfusion, which may predispose patients to the development of CVS—until recently considered a major contributor to delayed cerebral ischemia^18^. Furthermore, higher platelet activation was associated with CVS, DCI, and mortality after SAH^15,19^. Therefore, the role of PF4 as a marker of platelet reactivity suggests its potential utility in predicting adverse cerebrovascular events after SAH. In parallel, stromal cell-derived factor (SDF) has attracted attention for its multifaceted role in tissue repair, angiogenesis, and chemotaxis^20,21^. SDF has been suggested to orchestrate compensatory mechanisms in response to vascular injury and inflammation^21,22^ potentially mitigating the extent of brain tissue damage^23,24^. However, its association with clinical outcomes remains largely unknown.

Given the critical need for early and accurate biomarkers in SAH, the present study aims to elucidate the predictive value of PF4 and SDF levels in the context of SAH-related complications and mortality. Both PF4 and SDF are blood biomarkers that are easy to quantify and can serve as diagnostic markers. Early diagnostic and prognostic blood biomarkers are critical to guide clinicians in making timely critical decisions in post-aSAH management of patients. By investigating these associations, our work seeks to enhance the understanding of the inflammatory and hemostatic pathways that drive secondary injury following SAH and to identify potential biomarkers for clinical risk assessment and therapeutic intervention.

## Methods

### Study design, ethical approval and population

Between 2020 and 2022, we prospectively enrolled 63 patients with aneurysmal subarachnoid hemorrhage. The study was approved by the ethics committee of the Medical Faculty of Heinrich-Heine-University, Germany (Study-Nr.: 2019-787-bio). The relevant regulations and guidelines were followed for conducting the study. Written informed consent was obtained from all patients. When patients were unable to provide consent themselves, it was secured from their authorized proxy.

For the study, demographic data, WFN score, treatment modality (clipping/coiling), EVD placement, CVS, DCI clinical, DCI radiological, mRS and mortality were collected. PF4 and SDF were considered independent (predictor) variables, and day of death (in case of death), cerebral vasospasm (CVS, binary), and mean transit time (MTT) dependent variables (outcome). As the exact time point of death was not available, the day of death was summarized into six ordinal categories: death in the first 72 h, after 72 h and in the first 7 days, after 7 days and in the first 30 days, after 30 and up to 90 days, after 90 days, and ‘no death’. No patients belonged to the first category (death in the first 72 h).

### Definition of outcomes

Cerebral vasospasm was defined as arterial narrowing on CT angiography (CTA) in combination with a prolonged mean transit time (MTT > 4 s) on CT perfusion (CTP) in patients with clinical deterioration not explained by other causes. In selected cases, transcranial Doppler sonography (TCD) was performed as a supportive tool; a mean flow velocity > 120 cm/s in the middle cerebral artery or a Lindegaard Index > 3 was considered suspicious for vasospasm. In all cases meeting the above criteria, digital subtraction angiography (DSA) was performed to confirm the diagnosis.

Clinical delayed cerebral ischemia was defined as the occurrence of new focal neurological deficits or a decrease in consciousness after day 4 post-SAH, without other identifiable causes (e.g., hydrocephalus, seizures, infection).

Radiological DCI was defined as a new cerebral infarction detected on follow-up CT or MRI that was not present on initial imaging and could not be explained by procedural factors (e.g., EVD-related damage).

### CT perfusion imaging

CT perfusion studies were performed on a SOMATOM Definition Flash scanner (Siemens Healthineers, Germany) using standardized protocols. Imaging was conducted at days 0, 4, 7, and 11 post-aSAH. Perfusion maps were generated using the syngo CT 2012B software platform with the CT Neuro Perfusion module (Siemens Healthineers), which calculated mean transit time (MTT), cerebral blood flow (CBF), and cerebral blood volume (CBV) values semi-automatically.

For acquisitions dynamic imaging was conducted with a tube voltage of 80 kV and a tube current of 120 to 180 mA, resulting in an effective tube load of 120–180 mAs. Images were reconstructed with a slice thickness of 10 mm using the H20s convolution kernel.

The region of interest (ROI) was manually selected by two experienced neuroradiologists who were blinded to the clinical data. Interobserver variability was minimized through consensus review. MTT was expressed in seconds and served as a surrogate marker of cerebral perfusion.

### Serum collection

Blood samples were obtained from patients within 24 h of admission. The serum was separated by centrifuging the blood at 2000 relative centrifugal force for 10 min and was immediately stored at − 80 °C, until further use.

### Enzyme-linked immune assay (ELISA)

To quantify PF4 and SDF, the commercially available ELISA kits for PF4 (Bio-Techne, DY795) and SDF (Bio-Techne, DY350-05) were utilized, with the DuoSet ELISA Ancillary Reagent Kit 2 (Bio-Techne, DY008B) used for all assays. Prior to the procedures, a serum sample was serially diluted using a geometric series with the Reagent Dilution Buffer to determine the optimal dilution for each kit. The PF4 ELISA was conducted according to the manufacturer’s instructions using a 1:256 serum dilution, while the SDF ELISA was performed as specified in its manual with a 1:16 serum dilution. All dilutions were prepared using the Reagent Dilution Buffer.

### Data analysis

The primary endpoint was the presence of CVS. Secondary endpoints were MTT and the time point of death in non-survivors. The dependence of MTT and death timespan on PF4 and SDF values was modeled using linear regression, and the presence of CVS using logistic regression. The optimal cutoff for predicting CVS was found by maximizing the Youden index, and the predictor performance was quantified by the sensitivity, specificity, accuracy, and the area under the receiver operating characteristics curve (AUROC).

All computations were performed in Python (www.python.org), using NumPy^25^ SciPy^26^ and Stats models^27^ libraries.

Numerical variables were analyzed using a t-test to assess their association with dichotomous variables such as sex, age (< 60/≥60), WFNS, and (≤ 3/≥4). For the trichotomized outcome (mRS ≤ 2, 3 ≤ mRS ≤ 5, mRS = 6), one-way ANOVA was conducted separately. When the ANOVA indicated significant differences among outcome groups, post-hoc t-tests were performed to identify these differences. A detailed summary of the stratified values is provided in Tables 2, 3 and 4, and 5.

## Results

**Fig. 1:**
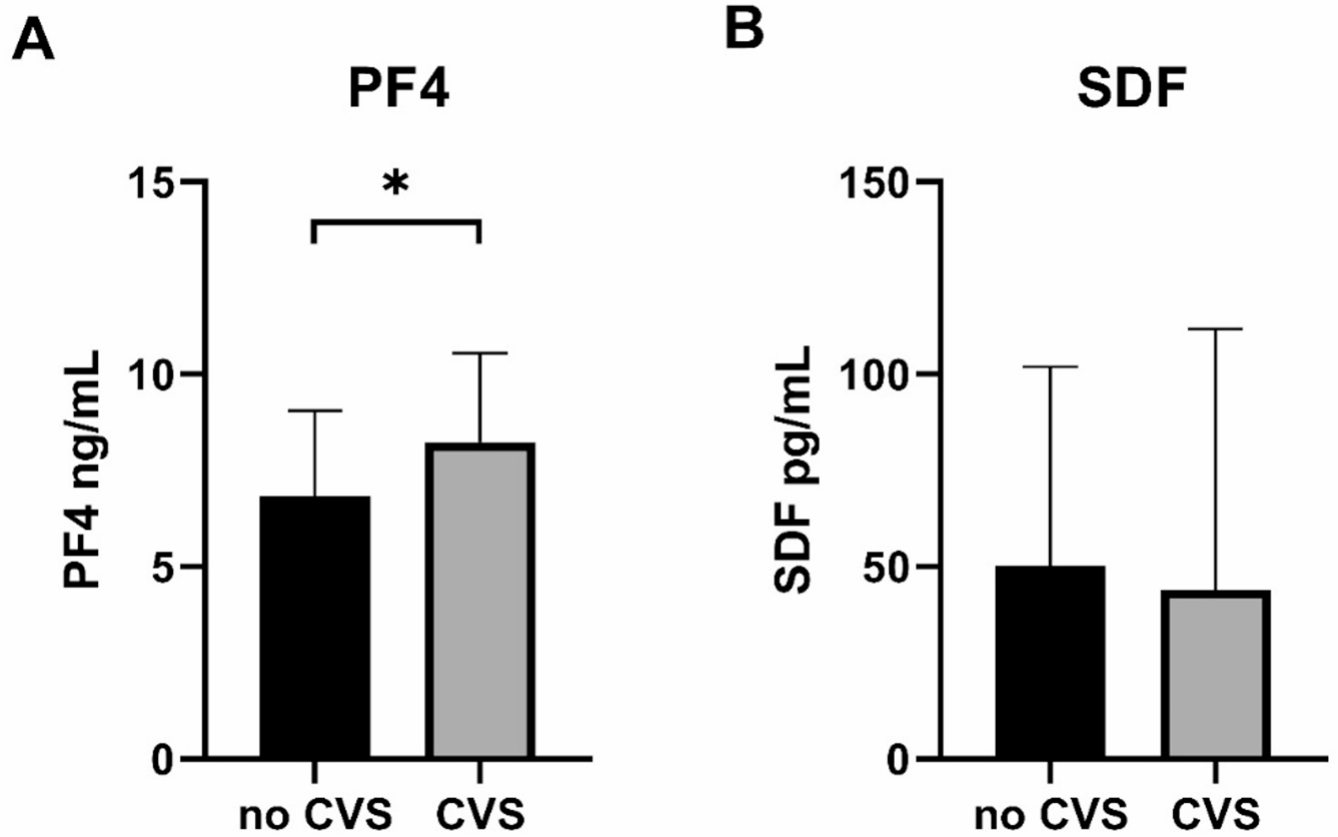
The comparison of PF4 and SDF levels in patients with and without CVS. **A** The PF4 concentration was significantly higher in the CVS group compared to the no CVS group (*p = 0.02). **B** No significant difference was observed between the two groups. The data was analyzed by performing a two-tailed t test.

The patient demographic is summarized in Table 1. The baseline characteristics of the patient cohort, including sex, age, and WFNS score, did not affect the serum concentrations of PF4 and SDF (Tables 2, 3, 4, 5). The PF4 levels were significantly higher in patients who developed CVS compared to those without CVS (no CVS: 6.8 ± 2.2 ng/mL vs. CVS: 8.3 ± 2.1 ng/mL, *P* = 0.02, Fig. 1A). One obvious outlier – an observation with SDF = 1742 pg, i.e. 20 inter-quartile ranges (IQR) above the third quartile (82 pg)—was discarded.

**Table 1:** Cohort demographics, total and by outcome group.

Levels	Total	mRS < = 2	3 < = mRS < = 5	mRS = 6	*p*
	n (%)	n (%)	n (%)	n (%)	
Sex					
M	20 (28%)	5 (22%)	11 (33%)	0 (0%)	0.2
W	51 (72%)	18 (78%)	22 (67%)	7 (100%)	
WFNS					
1	19 (30%)	13 (57%)	6 (18%)	0 (0%)	0.0001
2	6 (10%)	3 (13%)	3 (9%)	0 (0%)	
3	6 (10%)	2 (9%)	4 (12%)	0 (0%)	
4	13 (21%)	5 (22%)	8 (24%)	0 (0%)	
5	19 (30%)	0 (0%)	12 (36%)	7 (100%)	
Fisher					
2	1 (2%)	1 (5%)	0 (0%)	0 (0%)	0.06
3	16 (27%)	9 (47%)	5 (15%)	2 (29%)	
4	42 (71%)	9 (47%)	28 (85%)	5 (71%)	
CVS					
0	39 (67%)	15 (79%)	21 (64%)	3 (50%)	0.3
1	19 (33%)	4 (21%)	12 (36%)	3 (50%)	
VPS					
0	37 (63%)	17 (89%)	14 (42%)	6 (86%)	0.0001
1	21 (36%)	2 (11%)	19 (58%)	0 (0%)	
nA	1 (2%)	0 (0%)	0 (0%)	1 (14%)	
EVD					
0	9 (15%)	7 (37%)	2 (6%)	0 (0%)	0.006
1	50 (85%)	12 (63%)	31 (94%)	7 (100%)	
DCI_radio					
0	32 (55%)	18 (95%)	13 (39%)	1 (17%)	0.0001
1	26 (45%)	1 (5%)	20 (61%)	5 (83%)	
DCI_clinical					
0	28 (51%)	19 (100%)	8 (24%)	1 (33%)	0.0001
1	27 (49%)	0 (0%)	25 (76%)	2 (67%)	
Clipping					
0	17 28%)	5 (25%)	11 (33%)	1 (14%)	0.5
1	43 (72%)	15 (75%)	22 (67%)	6 (86%)	

**Table 2:** ELISA values stratified by sex.

Variable	Total	Sex = M	Sex = F	*p*
	Mean (sd)	Mean (sd)	Mean (sd)	
PF4 (ng)	6.9 (2.3)	6.8 (2.6)	6.9 (2.2)	0.9
SDF (pg)	59 (74)	69 (71)	48 (57)	0.2

**Table 3:** ELISA values stratified by age.

Variable	Total	Age < 60	Age > = 60	*p*
	Mean (sd)	Mean (sd)	Mean (sd)	
PF4 (ng)	6.9 (2.3)	7.0 (2.5)	6.7 (1.9)	0.6
SDF (pg)	59 (74)	65 (80)	44 (57)	0.3

**Table 4:** ELISA values stratified by initial status.

Variable	Total	WFNS < = 3	WFNS > = 4	*p*
	Mean (sd)	Mean (sd)	Mean (sd)	
PF4 (ng)	6.9 (2.3)	6.8 (2.2)	7.2 (2.5)	0.4
SDF (pg)	59 (74)	55 (79)	64 (67)	0.6

**Table 5:** Age onset, MTT and ELISA values stratified by outcome.

Variable	Total	mRS < = 2	3 < = mRS < = 5	mRS = 6	*p*
	Mean (sd)	Mean (sd)	Mean (sd)	Mean (sd)	
Age onset	54 (12)	52 (12)	54 (11)	63 (9)	0.08
MTT	3.87 (0.82)	3.78 (0.71)	3.95 (0.86)	3.77 (0.96)	0.8
PF4 (ng)	6.9 (2.3)	7.2 (2.2)	7.2 (2.5)	6.5 (1.8)	0.8
SDF (pg)	59 (74)	42 (42)	62 (67)	26 (27)	0.2

The SDF concentrations did not differ between the two groups (no CVS: 50 ± 52 pg/mL vs. CVS: 44 ± 70 pg/mL, *p* = 0.7, Fig. 1B).

**Fig. 2:**
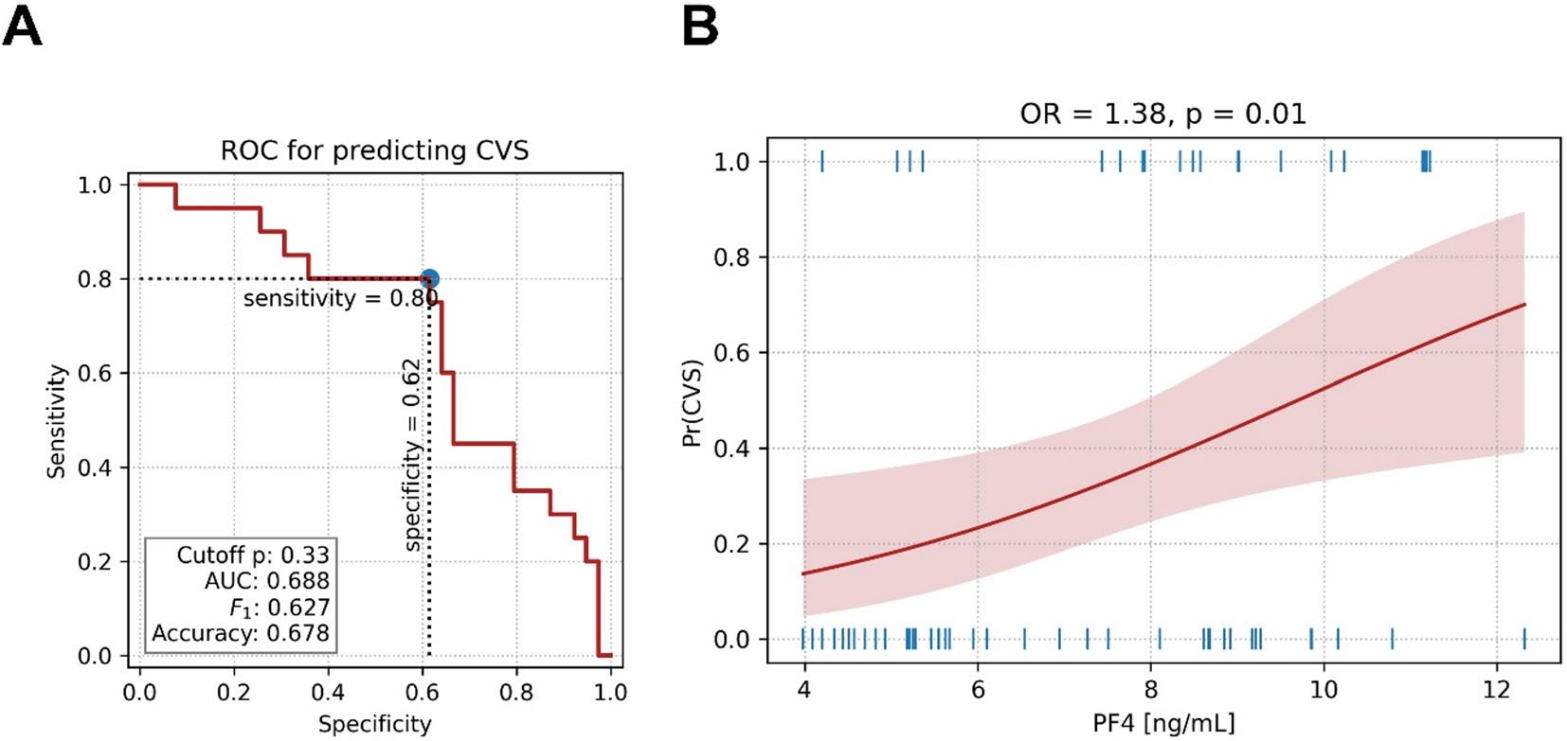
Predictive analysis for cerebral vasospasm (CVS) using ROC curve and PF4 logistic regression model. **A** The ROC curve evaluated the performance of the predictive model, yielding an AUC of 0.688, which indicated moderate discrimination. An optimal cutoff probability of 0.33 provided a sensitivity of 80%, a specificity of 62%, an F₁-score of 0.627, and an overall accuracy of 67.8%. **B** The logistic regression model of PF4 predicting CVS illustrated a significant positive association between PF4 levels and the likelihood of developing CVS. Precisely, each 1 ng/mL increase in PF4 corresponded to a 38% increase in the odds of CVS (OR = 1.38, *p* = 0.01). The red curve depicts the fitted logistic regression model, the shaded area indicates the 95% confidence interval, and the blue tick marks represent individual data points for CVS and non-CVS cases.

In the further analysis, the predictive potential of PF4 for CVS was analyzed, which revealed that PF4 levels exhibited moderate discriminatory power for predicting CVS, as evidenced by a receiver operating characteristic curve with an AUC of 0.688 (Fig. 2A). An optimal cutoff probability of 0.33—determined by maximizing the Youden index—yielded a sensitivity of 80%, a specificity of 62%, an overall accuracy of 67.8%, and an F₁-score of 0.627. Complementing these findings, logistic regression analysis demonstrated that each 1 ng/mL increase in PF4 levels was associated with a 38% increase in the odds of developing CVS (OR = 1.38; 95% CI: 1.07–1.79; *p* = 0.01, Fig. 2B), underscoring the potential of PF4 as a robust predictor of CVS. Additionally, in our study the analysis showed *p* = 0.063 value for association of PF4 with DCI.

**Fig. 3:**
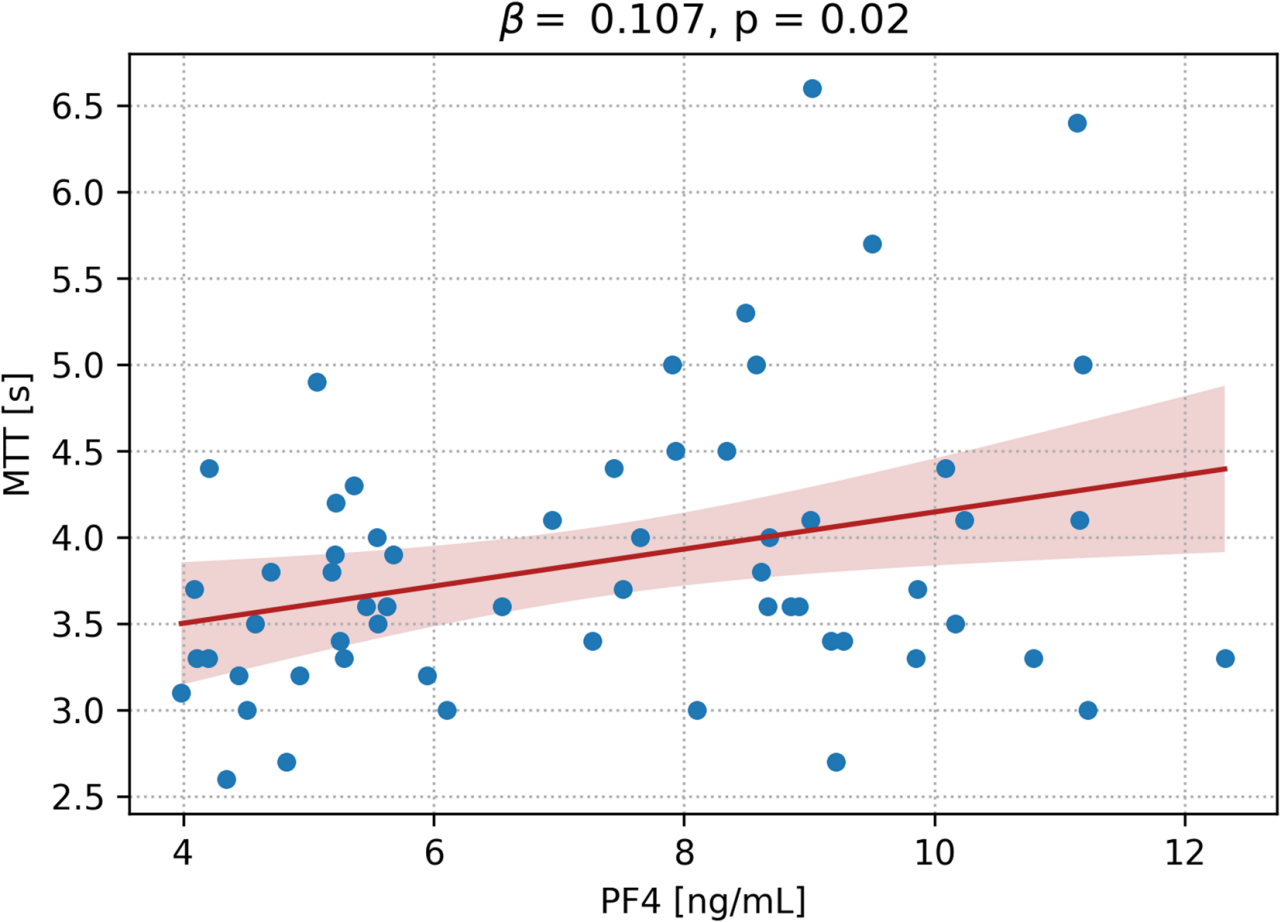
Association between PF4 Levels and Mean Transit Time (MTT). The scatter plot with a fitted regression line demonstrates a significant positive correlation between PF4 levels and MTT (β = 0.107, *p* = 0.02), suggesting that increased PF4 is associated with prolonged MTT. The red line indicates the regression fit, and the shaded region represents the 95% confidence interval.

The highest recorded MTT value across all time points (Day 0, Day 4, Day 7, Day 11) was used in the regression analysis to reflect the maximum degree of perfusion impairment. The scatter plot with regression analysis showed that higher PF4 levels were significantly associated with longer mean transit times (Fig. 3). The regression coefficient was β = 0.107 per ng/mL (95% CI: 0.02–0.19; *p* = 0.02), indicating that elevated PF4 levels correlated with impaired cerebral perfusion as reflected by increased MTT.

**Fig. 4:**
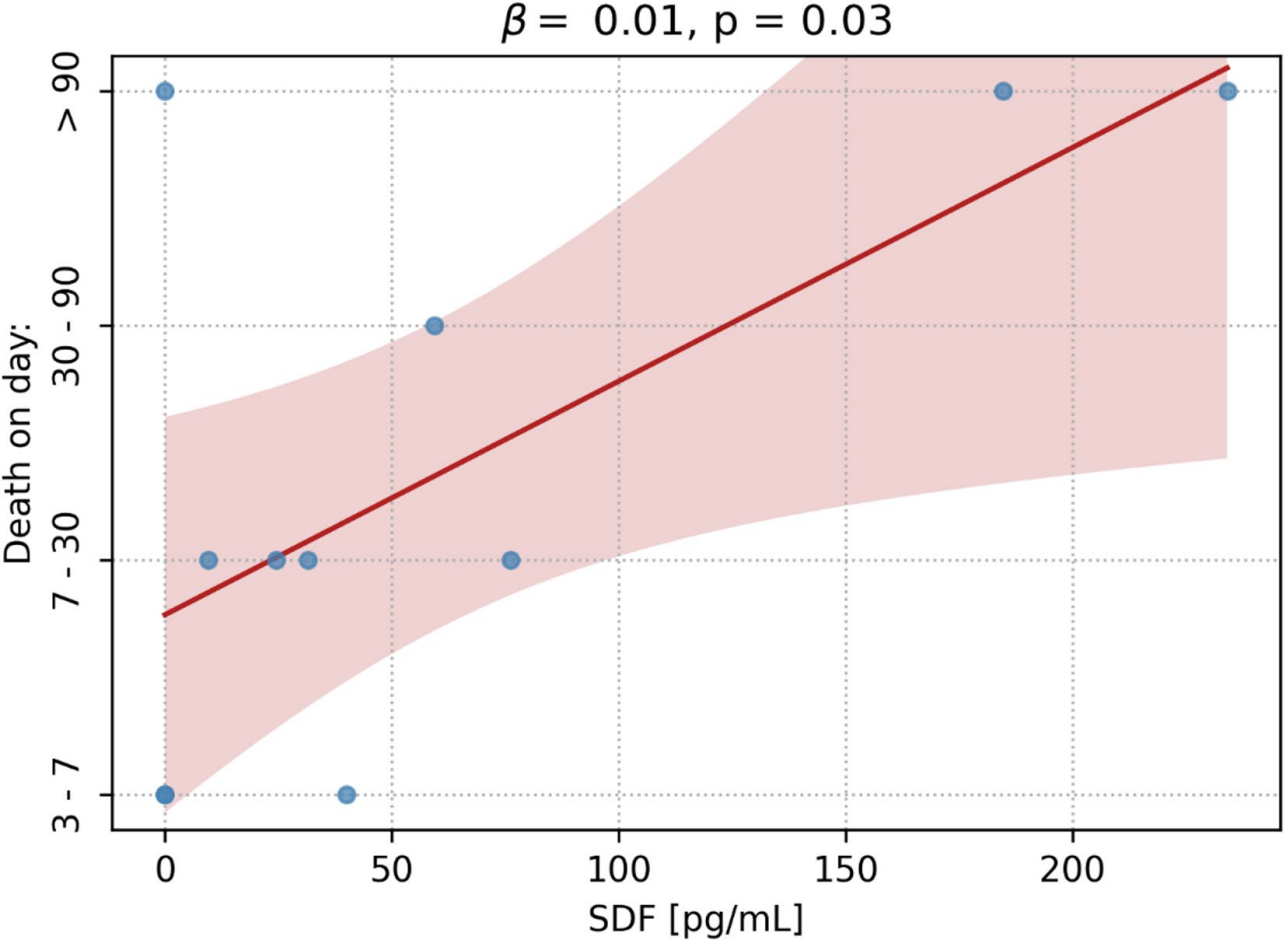
Linear Relationship between SDF Levels and Time to Death. A linear regression analysis showed a significant positive association between SDF levels and the day of death (β = 0.01, *p* = 0.03), indicating that higher SDF concentrations are linked to later mortality. The red line represents the fitted regression model, with the shaded area denoting the 95% confidence interval.

Among non-surviving patients, linear regression analysis demonstrated a statistically significant positive association between SDF levels and the day of death. The regression coefficient was β = 0.01 per pg/mL (95% CI: 0.001–0.019; *p* = 0.03, Fig. 4), suggesting that higher SDF levels are negatively correlated with in-hospital mortality.

## Discussion

The current study highlights the potential of PF4 and SDF as biomarkers for SAH-associated complications, particularly CVS and in-hospital mortality.

Our findings demonstrate that elevated PF4 levels were significantly increased in patients with CVS (Fig. 1A) and were significantly associated with an increased risk of CVS, as evidenced by the ROC curve analysis (AUC = 0.688) and logistic regression results indicating that each 1 ng/mL increase in PF4 corresponded to a 38% increase in the odds of developing CVS (Fig. 2). Moreover, the association between higher PF4 levels and prolonged mean transit times (Fig. 3) further suggested that PF4 may reflect impaired cerebral perfusion, potentially due to its role in platelet activation and inflammatory cascades^28^ following SAH^5,13,14^. While PF4 levels correlated with prolonged MTT, it is noteworthy that MTT is a perfusion parameter rather than a direct diagnostic marker of angiographic vasospasm. As suggested by Vergouwen and colleagues, DCI and cerebral infarction represent more definitive and clinically meaningful endpoints in aSAH^29^. In our cohort, PF4 levels demonstrated a trend toward association with radiological DCI (*p* = 0.063). Therefore our findings should be interpreted as associations with perfusion impairment, which mostely reflect angiographic vasospasm beyond a certain cut off. PF4, a chemokine stored in platelet α-granules, is released upon platelet activation—a common event after SAH and is associated with post-SAH complications and poor outcomes^15,19^. Once released, PF4 binds to glycosaminoglycans on the endothelium^28^increasing endothelial permeability, triggering a pro-inflammatory state, and promoting microthrombus formation^17,28^ which together contribute to vasoconstriction and microcirculatory impairment. The resulting inflammatory milieu and microthrombotic^17^ events have been suggested to contribute to the development of CVS^5,13,19^—previously considered a leading cause of delayed cerebral ischemia in SAH patients^18^. The current data corroborate earlier reports linking platelet activation with vascular dysfunction^7^ and post-SAH complications^15,19^ thereby reinforcing the notion that PF4 not only serves as a marker of platelet reactivity but also as an indicator of microcirculatory impairment.

In addition to PF4, our analysis of SDF levels among non-surviving patients revealed a positive correlation with the day of death. Specifically, higher SDF levels were associated with a delayed occurrence of death (Fig. 4). SDF, also known as CXCL12, plays a pivotal role in suppressing inflammation, chemotaxis, angiogenesis, and tissue repair by mobilizing endothelial progenitor cells to sites of vascular injury^20,22^. Moreover, SDF can exert anti-inflammatory^22,24^ anti-apoptotic^23,24^ and antioxidant effects by inducing HO-1 expression^20^. Its elevated levels may reflect an activated reparative response to the extensive vascular injury^21^ inflammation^22^ and brain tissue damage^24^ initiated by SAH^13^. However, despite these compensatory mechanisms^21^ persistently high SDF concentrations may also indicate the severity of the underlying pathophysiological processes that ultimately culminate in a fatal outcome.

Together, these findings underscore the multifaceted role of inflammatory and platelet-derived mediators in the progression of SAH-related complications^4,13,19^. PF4 emerges as a robust prognostic biomarker of CVS and impaired cerebral perfusion, suggesting that it could serve as a valuable biomarker for early risk stratification. Concurrently, the association between SDF levels and the timing of death in non-survivors points to a complex interplay between its protective^20–24^ and deleterious^30^ responses following SAH. This dual role of SDF^20–24,30^ may provide insights into the mechanisms that delay, but do not ultimately prevent, the cascade leading to death. Future studies should focus on validating these findings in larger, multicentric cohorts and further elucidating the molecular pathways involved, with the goal of developing targeted interventions that can modulate these biomarkers to improve clinical outcomes.

### Limitations

Despite these promising results, our study has several limitations. The observational design, single-centre study, and the relatively small sample size may limit the generalizability of the findings. Additionally, the use of a single time point for biomarker measurement within 24 h of admission may not capture temporal biomarker dynamics. Moreover, while the associations identified here are statistically significant, the underlying biological mechanisms remain to be fully elucidated. Future studies with larger cohorts and mechanistic investigations are warranted to validate these biomarkers and to explore potential therapeutic interventions aimed at modulating PF4 and SDF pathways.

## Conclusion

Our findings contribute to the growing body of evidence that implicates inflammatory and platelet activation pathways in the pathogenesis of CVS and poor outcomes following SAH. By identifying PF4 and SDF as potential prognostic biomarkers, this study lays the groundwork for future research aimed at improving risk stratification and tailoring therapeutic strategies to mitigate the devastating consequences of SAH.

## Data Availability

The datasets analyzed during the current study are available from the corresponding author on reasonable request.
